# Effect of Abscisic Acid on Accumulation of Five Active Components in Root of *Glycyrrhiza uralensis*

**DOI:** 10.3390/molecules22111982

**Published:** 2017-11-15

**Authors:** Jing Qiao, Zuliang Luo, Yanpeng Li, Guangxi Ren, Chunsheng Liu, Xiaojun Ma

**Affiliations:** 1 Institute of Medicinal Plant Development, Chinese Academy of Medical Sciences, Beijing 100193, China; qiaojing_happy@126.com (J.Q.); zuliangluo@163.com (Z.L.); 2 College of Traditional Chinese Medicine, Beijing University of Chinese Medicine, No. 6 Wangjing Zhonghuan Road, Beijing 100102, China; maidongxue@126.com (Y.L.); renguangxiabc@163.com (G.R.); max_liucs@263.net (C.L.)

**Keywords:** *Glycyrrhiza uralensis*, phytohormones, abscisic acid, active components, glycyrrhizin

## Abstract

Licorice is one of the most generally used herbal medicines in the world; however, wild licorice resources have decreased drastically. Cultivated *Glycyrrhiza uralensis* Fischer are the main source of licorice at present, but the content of main active components in cultivated *G. uralensis* are lower than in wild *G. uralensis*. Therefore, the production of high-quality cultivated *G. uralensis* is an urgent issue for the research and production fields. In this study, the content of five active components and seven endogenous phytohormones in cultivated *G. uralensis* (two-year-old) were determined by high-performance liquid chromatography (HPLC) and enzyme-linked immunosorbent assay (ELISA), respectively. Furthermore, different concentrations (25–200 mg/L) of exogenous abscisic acid (ABA) were sprayed on the leaves of *G. uralensis* in the fast growing period. Results showed that ABA, zeatin riboside (ZR), and dihydrozeatin riboside (DHZR) had strong correlation with active components. In addition, the content of five active components increased remarkably after ABA treatment. Our results indicate that ABA is significantly related to the accumulation of active components in *G. uralensis*, and the application of exogenous ABA at the proper concentration is able to promote the accumulation of main components in *G. uralensis*.

## 1. Introduction

**Figure 1:**
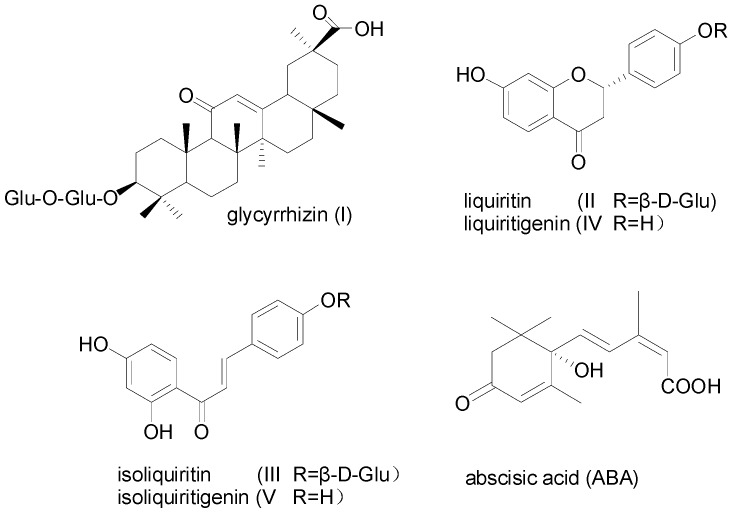
Chemical Structures of glycyrrhizin (**I**), liquiritin (**II**), isoliquiritin (**III**), liquiritigenin (**IV**), isoliquiritigenin (**V**), and abscisic acid (ABA).

The sources for licorice are the radix and rhizome of *Glycyrrhiza uralensis* Fisch. or *Glycyrrhiza. inflata* Bat. or *Glycyrrhiza. glabra* L. (Fabaceae Family) [1], and they have long been used as demulcents and expectorants in eastern countries, as well as flavoring and sweating agents in both Eastern and Western countries [2]. At present, glycyrrhizin (**I**) (Figure 1), an important bioactive triterpenoid saponin from the root of *G. uralensis*, is clinically used in treatments of hyperlipaemia, atherosclerosis, viral diseases, and allergic inflammation such as chronic hepatitis and atopic dermatitis [3], and is also commercially available as a sweetening and flavoring agent worldwide in the food, tobacco, and beer industries, since its sweetness is 150 times higher than that of sucrose [4,5]. Moreover, multiple flavonoids isolated from the root of *G. uralensis*, including liquiritin (**II**), isoliquiritin (**III**) [6], liquiritigenin (**IV**) [7], and isoliquiritigenin (**V**), etc. [8] have showed various bioactivities, such as anti-depression, liver protection, anti-inflammatory, anti-bacterial, and anti-cancer [9,10].

Wild *G. uralensis* has been the main source of licorice for decades. Now, over-harvest has gradually exhausted the wild resources, and cultivated *G. uralensis* are increasingly becoming an alternative source of *G. uralensis*. However, its quality is unstable [11]. For example, as the main active components, the content of compound **I** is lower in cultivated *G. uralensis* than in wild ones [12]. Therefore, the production of high-quality uralensis is an urgent issue for research and in the field. *G. uralensis* usually grows in arid and semiarid desert grassland, desert edges, and hilly areas. Because of this wild environment, *G. uralensis* may suffer stresses such as drought, salt, and high or low temperature. Previous study has demonstrated that there is a negative correlation between the accumulation of secondary metabolism in the medicinal plant and the environmental condition [13]. Li et al. demonstrated that a minor water deficit can increase the yield of active compounds in root of *G. uralensis* without negative effect on root growth [14]. Plants develop a wide range of adaptive mechanisms to meet these adverse conditions, including an adjustment of growth and development brought about by changes in stomatal activity; especially, the level of abscisic acid (ABA) changes under drought stress [15].

Abscisic acid (Figure 1) is a plant growth regulator that exerts prominent roles in plants in response to environmental stresses [16,17]. Many studies have suggested that exogenous ABA positively influences phytochemical content in selected plants. It has been demonstrated that the exogenous application of ABA increases the accumulation of phenolics in muscadine grapes [18]. Li et al. reported that ABA significantly increased the content of total phenolics and total anthocyanins in red lettuce [19]. These studies suggest that ABA plays a significant role in triggering the flavonoid biosynthetic pathway. Most studies investigating the effect of exogenous ABA application have been conducted in fruits and vegetables. However, the effect of ABA on medical plants such as *G. uralensis* has not been investigated. We hypothesized that the exogenous application of ABA may positively impact the accumulation of triterpenoid saponins and flavonoids in *G. uralensis*. The present study was designed to test these hypotheses by evaluating the effect of ABA on the morphological characters and accumulation of secondary metabolism in *G. uralensis*.

## 2. Results

### 2.1. The Determination of Active Components and Phytohormones in Root of *G. uralensis*

The contents of five kinds of active components and seven types of phytohormones in each plant are listed in Table 1. The contents of active components and phytohormones varied widely.

Among the 25 plants, the mean content of compound **I** was 2.11% and ranged from 0.82% to 3.46%. Compound **II** content ranged from 0.50% to 2.89% with a mean value of 1.30%, while compound **III** levels ranged from 0.70% to 4.83% (mean value was 2.21%). The amount of compounds **I**, **II** and **III** in the root of *G. uralensis* was much higher than compounds **IV** and **V**. The average values of compounds **IV** and **V** were 0.10% and 0.02%, respectively. The results showed that the contents of active components are significantly different between the different plants of *G. uralensis*.

During the analysis of *G. uralensis*, the presence of seven different phytohormones was also recorded. Among the 25 plants, the ABA content ranged from 37.59 to 92.04 ng/g with a mean value of 58.88 ng/g fw. The mean content of methyl jasmonate (MeJA) was 31.74 ng/g, and ranged from 14.40 to 55.95 ng/g fw. Among these, the levels of ABA and MeJA were much higher than that of any other phytohormone. In the root of *G. uralensis*, the average content of ABA was 6.68-fold, 8.02-fold, 6.13-fold, 5.68-fold, and 6.93-fold higher than zeatin riboside (ZR), indolepropionic acid (iPA), dihydrozeatin riboside (DHZR), gibberellic acid 3 (GA_3_), and brassinolide (BR), respectively. The average values of ZR, iPA, DHZR, GA_3_, and BR were 8.82, 7.34, 9.61, 10.37, and 8.50 ng/g fw, respectively.

**Figure 2:**
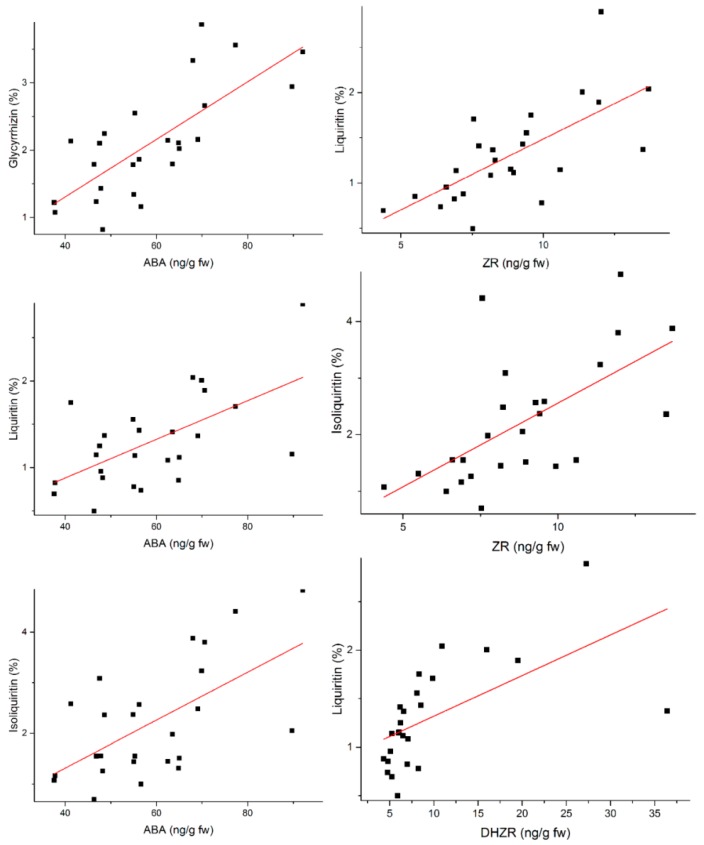
Scatter plot of the correlation between active compounds and phytohormones.

Correlation coefficients between five active components and seven phytohormones were analyzed using Pearson’s correlation analysis. Correlation analyses (Table 2) indicated that ABA content had significant positive correlation with the content of compounds **I**, **ΙΙ**, and **ΙΙΙ** (*r* = 0.746, 0.601, and 0.609, respectively) at the 0.01 level. ZR was significantly and positively correlated with compounds **I** and **ΙΙ** (*r* = 0.693 and 0.625) at the 0.01 level. DHZR had a positive and statistically significant correlation with the content of compound **ΙΙ** at the 0.01 level, *r* = 0.600. Figure 2 provides a scatter plot showing the positive correlation between active compounds and phytohormones (Spearman correlation *r* > 0.6, *p* < 0.01). In addition, GA_3_ had a positive correlation with compounds **I** and **ΙΙ** (*r* = 0.560 and 0.519) at the 0.01 level, and compound **ΙΙΙ** (*r* = 0.464) at the 0.05 level. MeJA and compound **ΙΙΙ**, ZR and compound **I** were also positively correlated at the 0.01 level—*r* = 0.506 and 0.519, respectively. However, there was no significant correlation between iPA and five kinds of active components. Additionally, the correlation was not significant between BR and active components.

### 2.2. Morphological Characteristics and the Content of Five Active Components in Root of G. uralensis Affected by Exogenous ABA

There was no significant difference in plant height, stem diameter, root diameter, or root weight in the control and ABA treated samples of *G. uralensis* from first sampling to last sampling (Table S1). However, during the first and second sampling periods, there was a significant increase in root length in 50 mg/L ABA-treated samples. Analysis of morphological characteristics of all *G. uralensis* plants revealed that the plants grew fastest before August and generally had steady growth until the last sampling.

**Figure 3:**
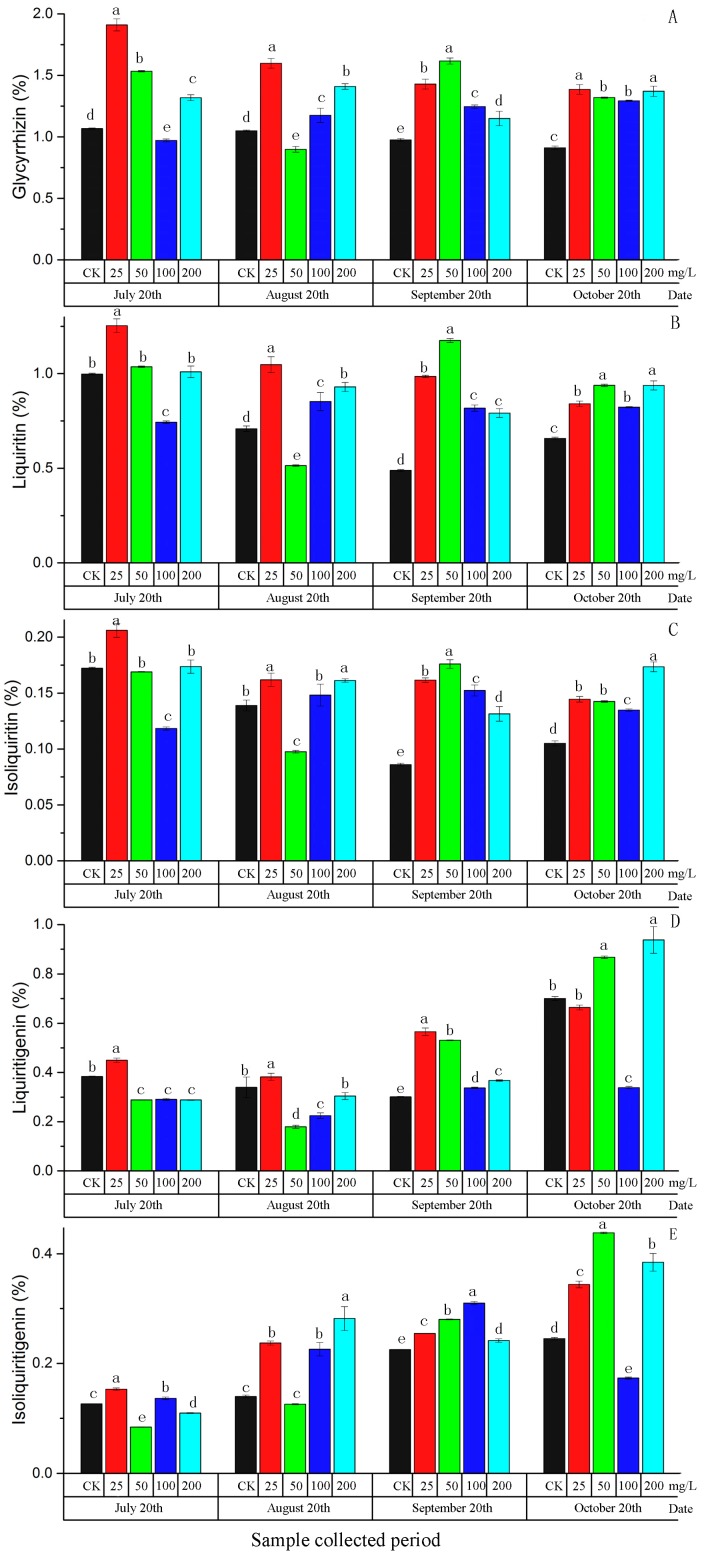
The effect of ABA on five active components in *G. uralensis* root. (**A**) glycyrrhizin (**I**), (**B**) liquiritin (**II**), (**C**) isoliquiritin (**III**), (**D**) liquiritigenin (**IV**), (**E**) isoliquiritigenin (**V**). Different letters followed by mean ± standard error indicate significant differences at *p* < 0.05. CK, Control solution contained Tween 20 without ABA.

As shown in Figure 3, the content of five active components in root of *G. uralensis* treated with different concentrations of ABA showed obvious differences (Table S2). The results indicated that the low concentrations could affect active components content for a shorter time than the higher concentrations.

The content of compound **I** in most samples of *G. uralensis* treated with ABA was higher than control in each sampling period. ABA at 25 mg/L greatly improved the content of compound **I** in all four sampling periods, and the difference between treated plants and control was significant. The contents increased by 78.96%, 52.28%, 46.42% and 52.01%, respectively. Similar to compound **I**, the content of compound **II** in *G. uralensis* treated with different concentrations of ABA were obviously different. The content of compound **II** in samples treated with ABA at 25 mg/L were improved and showed a significant difference compared with control in the four sampling periods. The increase for each of the four sampling periods were 25.61%, 47.80%, 101.62% and 27.86%, respectively.

At harvest time, five active components in most samples of *G. uralensis* treated with ABA obviously increased compared to control. Contents of compounds **I**, **II**, **III**, **IV**, **V** were significantly higher in *G. uralensis* treated with 200 mg/L ABA compared to control, and the increase rates were 50.38%, 42.61%, 65.12%, 22.13% and 56.98%, respectively. Moreover, the content of five components in *G. uralensis* treated with ABA at 25 and 50 mg/L also showed differences compared with the control, but the differences were not as obvious as those treated with 200 mg/L. In general, the exogenous application of ABA with four different concentrations significantly enhanced the content of compounds **I**, **II** and **III**, whereas compounds **IV** and **V** were decreased after treatment with 100 mg/L. Therefore, exogenous treatment with ABA at 200 mg/L in mid-to-late June could significantly promote the accumulation of five active components in *G. uralensis*.

## 3. Discussion

It is generally accepted that the production and distribution of the active components in medical plants is regulated by genetic, growth years, environmental, physiological, and chemical factors. It has been found that the growth of *G. uralensis* can be affected by temperature, rainfall, soil, water, or salt stress, and ABA exerts prominent roles in plants in response to these environmental stresses [20,21,22]. However, the effects of ABA on *G. uralensis* remain unstudied. The aims of the present study were to identify relationships between active components and phytohormones and to observe the changes in active component contents during root development after ABA application.

In root of *G. uralensis*, the most important compounds present are glycyrrhizin (**I**), liquiritin (**II**), isoliquiritin (**III**), liquiritigenin (**IV**), and isoliquiritigenin (**V**). Using the HPLC method developed in this study, we simultaneously determined the contents of five active components in *G. uralensis*. This method was successfully applied to analysis of the active components in *G. uralensis* treated by ABA. It is demonstrated that phytohormones are the essential players in the regulation of plant growth. Knowledge about their distribution in medicinal plant tissues is necessary for understanding the mechanisms of effective composition accumulation. Therefore, an efficient and simplified ELISA method for the simultaneous determination of seven phytohormones in *G. uralensis* was also developed. Results showed that ABA and the content of compounds **I**, **ΙΙ**, and **ΙΙΙ** showed a strong correlation at the 0.01 level (bilateral). Similar results were also observed by Daszkowskagolec et al., who found that there was a positive correlation of endogenous ABA and glycyrrhizic acid under drought stress [15]. In our study, the positive correlation between phytohormone level and active components content in the roots *G. uralensis* suggest that phytohormone may regulate the accumulation of active components.

**Figure 4:**
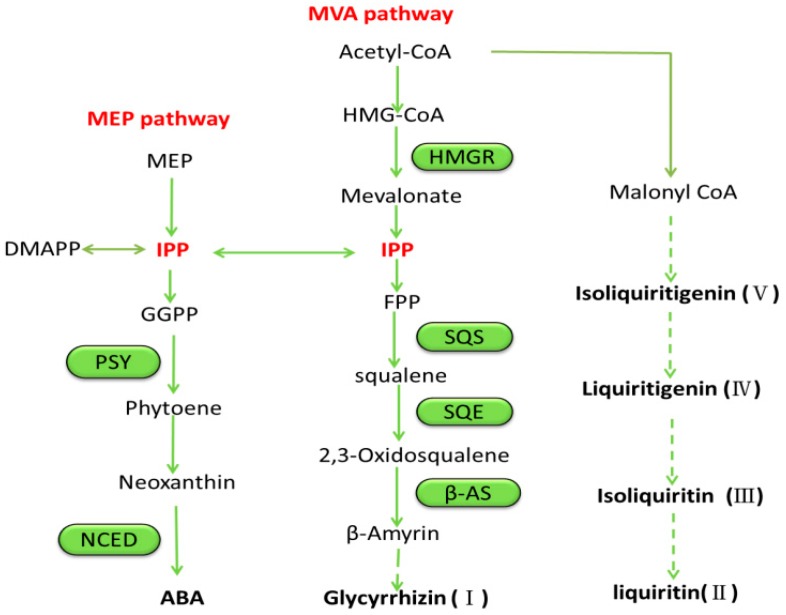
Biosynthesis pathway of ABA and five active components along with intermediates studied in this report. Biosynthesis of five active components via mevalonate (MVA) pathway, ABA biosynthesis via methylerythritol phosphate (MEP) pathway. HMG-CoA, 3-hydroxy-3-methyl glutaryl coenzyme A; DMAPP, dimethylallyl diphosphate; IPP, isopentenyl diphosphate; GGPP, geranylgeranyl pyrophosphate; FPP, farnesyl diphosphate; PSY, phytoene synthase; NCED, 9-*cis*-epoxycarotenoid dioxygenase; HMGR, HMG-CoA reductase; SQS, squalene synthase; SQE, squalene epoxidase; β-AS, beta-amyrin synthase.

ABA has been applied to increase accumulation of secondary metabolites and improve growth in the skin of vegetables and fruits. The concentrations used in this study were chosen based on those applied on other species [18,23,24,25]. In this study, the exogenous application of ABA significantly induced the accumulation of glycyrrhizin (**I**), liquiritin (**II**), isoliquiritin (**III**), liquiritigenin (**IV**), and isoliquiritigenin (**V**). A possible explanation is that these classes of secondary metabolites share a common synthetic pathway. Compound **I** is an effective component for quality control of *G. uralensis* according to the Chinese pharmacopoeia. Its biosynthesis occurs via the mevalonate (MVA) pathway, while ABA biosynthesis occurs via the methylerythritol phosphate (MEP) pathway. The two pathways intersect at isopentenyl diphosphate (IPP) or its isomer dimethylallyl diphosphate (DMAPP) (Figure 4). A feedback regulation mechanism may exist in the growth control of *G. uralensis*. When ABA increases under exogenous stimulus, the content of IPP or DMAPP may increase, and then it may lead to the increase of pharmaceutical ingredients via the other synthetic route for the same substrate they shared. This may be one way by which ABA regulates the synthesis of secondary metabolites. As for the flavonoids (compounds **II**, **III**, **IV**, **V**) in *G. uralensis*, previous study showed that ABA stimulated anthocyanin biosynthesis in crops because it induced the expression of several enzymes in the flavonoid biosynthetic pathway [26]. The same mechanism was verified in grapes [27]. This may be another mechanism by which ABA regulates the synthesis of secondary metabolites. Flavonoids are products of secondary metabolism in plants and are generated by a common phenylpropanoid pathway. Although these four flavonoid compounds share a common biosynthetic pathway, specific enzymes are involved in their synthesis, which may respond differently to exogenous ABA. Therefore, the results of this study need to be further explored from a functional gene level.

ABA plays an important role in adaptive responses to environmental stresses such as heat stress, water stress, and salt stress [16]. Plants accumulate endogenous ABA when subjected to environmental stresses. *G. uralensis* usually grows in arid and semiarid desert grassland. As a critical signal transduction compound, ABA subsequently upregulates the transcription of stress responsive genes to protect the plants [28]. In this study, a significant decrease in the content of active components in the control group was seen in September, which may be because of the excessive rainfall. However, same trend was not found in ABA-treated groups. We hypothesized that ABA could act as a stress factor to resist the decrease of secondary metabolites. In this study, upon exogenous ABA stimulation, plants can mobilize their defense mechanisms, and actively regulate the strength of the synthesis of secondary metabolites to improve the accumulation. Previous study showed that the contents of components **I** and **II** were positively correlated with temperature and negatively correlated with moisture [11]. In our study, the differences caused by other factors can be avoided because all plants of *G. uralensis* were planted and grown in same ecological environment.

In this study, the positive correlation between GA_3_, ZR, DHZR, MeJA, and active components in the roots suggest that these four phytohormones may also regulate the accumulation of active components. Li et al. demonstrated that GA_3_ at 40 mg/L applied in June significantly promoted the accumulation of glycyrrhizic acid in *G. uralensis* root [29]. However, whether applied GA_3_ can be used to increase the content of other active components remains unclear. Applications of ZR are in commercial industry and research regions are minor compared to other plant hormones. In addition to ABA, GA_3_, and ZR, other phytohormones, such as DHZR, MeJA, naphthaleneacetic acid (NAA), and BRs, have also been shown to play roles in plant growth and quality improvement. Additionally, hormone groups often interact with each other. Additional experiments of the application of ABA and other phytohormones have been planned in *G. uralensis*.

## 4. Materials and Methods

### 4.1. Chemicals

The reference standards of compounds **I** (>99.0% purity) and **II** (98.9% purity) were purchased from the Chinese Institute for the Control of Pharmaceutical and Biological Products (Beijing, China). Compounds **III**, **IV**, and **V** were obtained from Beijing Ding-guo Biotechnology CO., Ltd. (Beijing, China), with a purity of greater than 98%. HPLC-grade methanol, acetonitrile, phosphoric acid, and Tween 20 were products of Fischer Scientific Co. (Pittsburg, PA, USA). ABA was bought from Beijing Bio-Dee Biotechnology CO., Ltd. (Beijing, China).

### 4.2. Plant Materials and ABA Applications

Plant materials for content analysis, *G. uralensis* seeds were sowed on 1 May 2014, in the farm of Beijing University of Chinese Medicine (39°55′ N, 116°28′ E, Beijing, China) and harvested on 30 October 2015 by cutting the roots from plants. The plants were cultivated at an average temperature of 12.8 °C and an annual rainfall of 578.9 mm. Plant roots were collected at a fixed time, and root tips were cut and frozen in liquid nitrogen and saved at −80 °C for endogenous phytohormone analysis.

Plant materials for ABA spraying experiment, one-year old cultivated *G. uralensis* (320 plants) were collected from Gansu Province (Gansu, China) and transplanted into plastic pots (41 cm in height and 38 cm in diameter), eight plants per pot, filled with same volume of soil (containing organic matter (0.286%), alkali-hydrolyzable nitrogen (35.88 mg/kg), soil-available phosphorus (3.0 mg/kg), soil-available potassium (85.18 mg/kg) and CaCO_3_ (2.71%) in the farm of Beijing University of Chinese Medicine on 1 May 2015.

ABA was dissolved in water, and Tween 20 was added as a leaf wetting agent (315 μL/L) to obtain a concentration series (25, 50, 100 and 200 mg/L) of ABA solution. Control solution contained Tween 20 without ABA. According to spraying concentration, five groups were divided and there were three replications of each group (64 plants). The experiment was arranged in a randomized complete block design. The *G. uralensis* plants received foliar spray of ABA in different concentrations (0, 25, 50, 100, 200 mg/L). ABA application dates were chosen during the fast-growing period and to avoid rainfall and wind. The first foliar spray was carried out on 10 June 2015, using 125 mL per pot on the surface of the leaves using a hand-held garden sprayer. The second and third sprays were done two and four days later using the same volume. The root of *G. uralensis* was harvested four times on 20 July, 20 August, 20 September and 20 October to research the long-term dynamic changes of five active components and morphological characters.

Each *G. uralensis* root was weighed and measured. The root length, root diameter, root weight, plant height, and stem diameter were recorded to evaluate the growth status. A composite sample of each replication was dried (60 °C for 3 day) and powdered for solvent extraction.

### 4.3. HPLC Analysis

The whole dried roots were cut into small pieces and ground into fine powder. Dried samples (0.20 g) were extracted with 70% EtOH (50 mL) for 30 min under ultrasonication (250 W, 40 °C). Then, the solution was cooled to room temperature, adjusted with 70% EtOH to original volume, mixed, and passed through a membrane filter having 0.45 μm porosity. A volume of 10 μL was used for HPLC analysis.

HPLC analysis was performed with Agilent series 1200 equipment consisting of a G1311A pump, G1315B-Diode array detector, and G1316A column compartment. Samples were separated on an Agilent TC-C18 column (4.6 mm i.d. × 250 mm, 5 μm particle). The mobile phase was a gradient prepared from CH_3_CN (component A) and water containing 0.05% H_3_PO_4_ (component B), and the conditions used for gradient elution were: 0–8 min, 20% A; 8–30 min, 20–38% A; 30–42 min, 38–50% A; 42–50 min, 50–95% A. The flow rate was 1.0 mL/min. The wavelength conversion based on the maximum absorption peak was changed as follows: 0–15 min, 237 nm; 15–23 min, 365 nm; 23–30 min, 237 nm; 30–37 min, 370 nm; 37–45 min, 237 nm. The injection volume for all samples was 10 μL. The column was maintained at 30 °C during the analysis.

**Figure 5:**
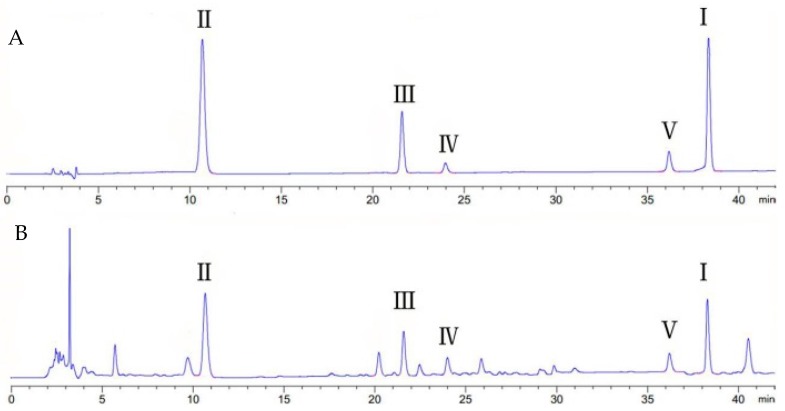
Chromatograms of five active components in the root of *G. uralensis*: (**A**) reference standard; (**B**) sample. **I**, glycyrrhizin; **ΙΙ**, liquiritin; **ΙΙΙ**, isoliquiritin; **ΙV**, liquiritigenin; **V**, isoliquiritigenin.

HPLC chromatograms of the standards of five main active components can be seen in Figure 5A, and the samples are shown in Figure 5B.

### 4.4. Phytohormone Measurements

The phytohormone (ZR, iPA, DHZR, GA_3_, MeJA, ABA, BR) concentrations in root tip were measured using the competitive enzyme-linked immunosorbent assay (ELISA) kit (China Agriculture University, Beijing, China). Approximately 0.50 g (fresh weight) of each root tip material was subjected to analysis. Root tip materials were first ground in liquid nitrogen, and then the powder was freeze-dried. The powder was extracted for 4 h at 4 °C in cold 80% methanol. The mixture was then centrifuged at 4000 r/min for 15 min and the supernatant was collected. The extraction was repeated on the pellet, and the supernatants were pooled. The pellet was washed a further three times in cold 80% methanol by vortexing and spinning, and the supernatant for each sample was pooled and dried down in a Speedy Vac until approximately 50 μL of liquid remained. TBS buffer (25 mM Tris–HCl pH 7.5, 100 mM NaCl, 1 mM MgCl_2_, 3 mM NaN_3_) was added to a final volume of 1.5 mL.

These extracts were diluted 10-fold in TBS and used in the ELISA according to the kit protocol. A standard curve of different ZR, iPA, DHZR, GA_3_, MeJA, ABA, and BR dilutions was constructed to calculate the concentrations. Phytohormone concentrations were calculated as ng per g. Each measurement was replicated three times.

### 4.5. Statistical Analysis

Data were expressed as mean ± standard deviation. IBM SPSS statistics software 22.0 was used for result analysis, including descriptive statistics of data, correlation analysis of factors, and regression.

## 5. Conclusions

In this study, the content of five active components and seven endogenous phytohormones in cultivated *G. uralensis* were determined by HPLC and ELISA, respectively. The results of Pearson correlation coefficient revealed a strong correlation between active components content and phytohormone level. Exogenous ABA application showed ABA can be a promising tool for the enhancement of triterpene saponins and flavonoid compounds without affecting the yield in *G. uralensis*. This study provides the basic data of ABA effect on the accumulation of active components in *G. uralensis*. ABA and other phytohormones should be employed in the further work to breed high-quality cultivated *G. uralensis.*

## Figures and Tables

**Table 1:** Variation in contents of five main active components and seven phytohormone levels in different plants of *G. uralensis*. ZR, zeatin riboside; iPA, indolepropionic acid; DHZR, dihydrozeatin riboside; GA**_3_**, gibberellic acid 3; MeJA, methyl jasmonate; BR, brassinolide.

Number	I	II	III	IV	V	ZR	iPA	DHZR	GA_3_	MeJA	ABA	BR
1	3.46	2.89	4.83	0.10	0.03	12.03	10.50	27.26	14.39	43.67	92.04	8.38
2	2.66	1.89	3.80	0.14	0.03	11.94	5.92	19.49	12.34	40.95	70.57	10.51
3	3.33	2.04	3.88	0.08	0.03	13.69	10.14	10.91	12.59	28.68	67.97	7.83
4	2.10	1.25	3.09	0.04	0.01	8.31	5.50	6.23	7.36	49.18	47.55	8.95
5	1.43	0.96	1.55	0.13	0.03	6.59	4.65	5.09	8.43	24.59	47.77	8.68
6	2.15	1.08	1.45	0.07	0.02	8.15	5.64	7.06	7.63	31.63	62.45	9.22
7	2.11	0.85	1.31	0.08	0.02	5.50	5.52	4.83	6.84	29.90	64.85	8.55
8	1.08	0.82	1.16	0.06	0.01	6.88	5.42	7.00	6.50	25.31	37.77	7.65
9	2.14	1.75	2.59	0.07	0.03	9.57	6.16	8.35	9.85	29.29	41.20	8.95
10	2.55	1.14	1.55	0.07	0.02	6.94	6.74	5.27	11.68	27.40	55.27	7.17
11	1.34	0.78	1.44	0.03	0.02	9.94	5.36	8.25	8.29	23.83	55.01	7.87
12	3.56	1.71	4.41	0.13	0.03	7.56	5.64	9.86	12.01	27.51	77.34	9.27
13	2.16	1.37	2.48	0.08	0.02	8.23	5.40	6.59	8.14	31.76	69.09	9.29
14	1.16	0.74	1.00	0.06	0.02	6.39	4.83	4.78	5.57	14.40	56.58	8.21
15	1.86	1.43	2.57	0.09	0.02	9.28	8.79	8.53	12.29	41.98	56.18	9.36
16	2.94	1.15	2.05	0.10	0.02	8.85	5.58	6.06	6.97	27.28	89.69	9.20
17	0.82	0.88	1.26	0.04	0.01	7.19	5.35	4.33	6.10	34.23	48.23	8.30
18	1.24	1.15	1.55	0.20	0.03	10.59	11.26	6.00	10.65	29.04	46.77	9.79
19	2.02	1.12	1.51	0.13	0.02	8.96	4.68	6.51	8.79	37.05	65.00	7.91
20	2.25	1.37	2.36	0.09	0.02	13.50	22.60	36.41	25.54	55.95	48.57	8.64
21	3.87	2.01	3.24	0.27	0.04	11.36	11.24	15.99	21.81	37.44	69.91	9.84
22	1.22	0.70	1.07	0.19	0.03	4.39	6.80	5.25	6.39	21.57	37.59	4.27
23	1.79	0.50	0.70	0.03	0.01	7.52	7.20	5.89	11.10	21.88	46.34	10.07
24	1.80	1.41	1.98	0.13	0.02	7.74	4.55	6.18	8.82	28.92	63.49	8.49
25	1.78	1.56	2.37	0.14	0.03	9.41	8.12	8.08	9.18	30.09	54.88	6.03

**Table 2:** Correlation between five active components and seven phytohormones in root of *G. uralensis* from 25 plants.

Pearson Coefficient	ZR	iPA	DHZR	GA_3_	MeJA	ABA	BR
**I**	0.519 **	0.258	0.460 *	0.560 **	0.343	0.746 **	0.318
**II**	0.693 **	0.325	0.600 **	0.519 **	0.496 *	0.601 **	0.209
**III**	0.625 **	0.253	0.552 **	0.464 *	0.506 **	0.609 **	0.271
**IV**	0.162	0.230	0.156	0.371	0.062	0.160	−0.042
**V**	0.374	0.232	0.286	0.402 *	−0.014	0.306	−0.043

Note: ** Means correlation at the 0.01 level (bilateral); * Means correlation at the 0.05 level (bilateral).

## Supplementary Materials

Supplementary materials are available online. Table S1: Average Plant height, Stem diameter, Root length, Root diameter and Root weight from control and ABA treated *G. uralensis*. Table S2: Accumulation of active components in root of *G. uralensis*.
