# Supplementary material for: Effect of Abscisic Acid on Accumulation of Five Active Components in Root of Glycyrrhiza uralensis
Source: Molecules. 2017 Nov 15;22(11):1982. doi: 10.3390/molecules22111982 (PMC6150281; doi:10.3390/molecules22111982)
Supplement: Supplementary file 1 [file molecules-22-01982-s001.pdf]

molecules

MDPI

Article

Effect of Abscisic Acid on Accumulation of Five Active Components in Root of *Glycyrrhiza uralensis*

Jing Qiao [1], Zuliang Luo [1], Yanpeng Li [2], Guangxi Ren [2], Chunsheng Liu [2] and Xiaojun Ma [1,*]

[1] Institute of Medicinal Plant Development, Chinese Academy of Medical Sciences, Beijing 100193, China; qiaojing_happy@126.com (J.Q.); zuliangluo@163.com (Z.L.)
[2] College of Traditional Chinese Medicine, Beijing University of Chinese Medicine, No. 6 Wangjing Zhonghuan Road, Beijing 100102, China; (Y.L.); renguangxiabc@163.com (G.R.); max_liucs@263.net (C.L.)
* Correspondence: mayixuan10@163.com; Tel.: +86-10-62819410

Table S1. Average plant height, stem diameter, root length, root diameter, and root weight from control and ABA-treated *G. uralensis*.

Harvest Time (Date)	Treatment (mg/L ABA)	Plant Height (cm)	Stem Diameter (mm)	Root Length (cm)	Root Diameter (mm)	Root Weight (g)
20 July	0	55.45 ± 11.60 a	3.02 ± 1.00 a	24.73 ± 4.60 ac	10.22 ± 2.63 a	12.65 ± 8.87 a
	25	55.31 ± 15.15 a	3.31 ± 0.99 a	28.46 ± 4.98 a	10.72 ± 2.58 a	13.64 ± 7.02 a
	50	52.83 ± 12.16 a	3.10 ± 0.69 a	33.67 ± 9.00 b	10.10 ± 2.23 a	12.13 ± 4.30 a
	100	59.9 ± 11.54 a	3.34 ± 0.97 a	30.91 ± 6.58 b	11.53 ± 2.06 a	14.45 ± 5.11 a
	200	57.79 ± 13.88 a	3.57 ± 0.77 a	28.93 ± 5.76 a	10.22 ± 3.74 a	14.24 ± 7.18 a
20 August	0	63.12 ± 18.10 a	2.59 ± 1.01 a	29.00 ± 5.62 bc	12.35 ± 3.72 a	26.02 ± 17.59 a
	25	62.00 ± 15.74 a	2.80 ± 1.24 a	29.25 ± 7.42 b	10.93 ± 2.86 a	13.65 ± 8.18 a
	50	70.63 ± 8.26 a	3.17 ± 1.03 a	34.13 ± 11.65 b	12.27 ± 2.87 a	18.41 ± 8.05 a
	100	68.36 ± 12.55 a	2.63 ± 0.68 a	37.25 ± 8.55 a	12.13 ± 2.59 a	19.93 ± 11.86 a
	200	65.92 ± 16.89 a	3.08 ± 1.12 a	29.17 ± 6.10 bc	11.78 ± 1.91 a	19.63 ± 7.92 a
20 September	0	56.30 ± 15.85 a	3.06 ± 1.35 a	30.71 ± 10.67 a	12.05 ± 2.99 a	21.86 ± 18.96 a
	25	63.30 ± 10.54 a	3.18 ± 0.59 a	27.60 ± 6.40 b	12.63 ± 3.05 b	23.86 ± 12.17 a
	50	66.33 ± 12.83 a	3.59 ± 0.56 a	28.56 ± 4.13 a	13.51 ± 1.69 a	26.24 ± 8.61 a
	100	58.43 ± 28.90 a	3.09 ± 0.94 a	29.57 ± 9.27 a	11.97 ± 2.91 a	18.73 ± 11.65 a
	200	63.11 ± 14.37 a	4.04 ± 1.17 a	30.22 ± 9.70 a	14.57 ± 3.09 a	28.88 ± 13.46 a
20 October	0	65.70 ± 18.73 a	3.56 ± 1.60 a	25.40 ± 5.44 a	14.25 ± 2.74 a	28.79 ± 13.38 a
	25	54.38 ± 21.00 a	2.61 ± 0.99 a	26.56 ± 4.19 a	12.66 ± 4.60 a	22.12 ± 18.78 a
	50	66.11 ± 14.73 a	3.56 ± 1.13 a	30.56 ± 8.95 a	13.54 ± 3.75 a	24.14 ± 15.13 a
	100	60.33 ± 14.69 a	3.22 ± 0.82 a	27.33 ± 6.10 a	12.02 ± 2.55 a	20.13 ± 9.11 a
	200	62.40 ± 12.76 a	3.27 ± 1.13 a	27.14 ± 5.40 a	12.88 ± 5.49 a	31.44 ± 26.25 b

Data are mean ± standard deviation for n = 16. Means with different letters denote significant differences ($p \leq 0.05$) per harvest date.

Table S2. Accumulation of active components in root of *G. uralensis*.

Harvest Time (Date)	Treatment (mg/L ABA)	I	II	III	IV	V
20 July	0	1.07 ± 0.01 d	1.00 ± 0.01 b	0.17 ± 0.00 b	0.38 ± 0.00 b	0.13 ± 0.00 c
	25	1.91 ± 0.05 a	1.25 ± 0.04 a	0.21 ± 0.01 a	0.45 ± 0.01 a	0.15 ± 0.00 a
	50	1.53 ± 0.01 b	1.04 ± 0.00 b	0.17 ± 0.00 b	0.29 ± 0.00 c	0.08 ± 0.00 e
	100	0.97 ± 0.01 e	0.74 ± 0.01 c	0.12 ± 0.00 c	0.29 ± 0.00 c	0.14 ± 0.00 b
	200	1.32 ± 0.02 c	1.01 ± 0.03 b	0.17 ± 0.01 b	0.29 ± 0.00 c	0.11 ± 0.00 d
20 August	0	1.05 ± 0.01 d	0.71 ± 0.02 d	0.14 ± 0.01 b	0.34 ± 0.04 b	0.14 ± 0.00 c
	25	1.60 ± 0.04 a	1.05 ± 0.04 a	0.16 ± 0.01 a	0.38 ± 0.02 a	0.24 ± 0.00 b
	50	0.90 ± 0.02 e	0.51 ± 0.00 e	0.10 ± 0.00 c	0.18 ± 0.01 d	0.13 ± 0.00 c
	100	1.18 ± 0.06 c	0.85 ± 0.05 c	0.15 ± 0.01 b	0.22 ± 0.01 c	0.23 ± 0.01 b
	200	1.41 ± 0.02 b	0.93 ± 0.02 b	0.16 ± 0.00 a	0.30 ± 0.01 b	0.28 ± 0.02 a
20 September	0	0.98 ± 0.01 e	0.49 ± 0.01 e	0.09 ± 0.01 e	0.30 ± 0.00 e	0.23 ± 0.00 e
	25	1.43 ± 0.04 b	0.99 ± 0.01 b	0.16 ± 0.02 b	0.57 ± 0.02 a	0.25 ± 0.00 c
	50	1.62 ± 0.03 a	1.18 ± 0.01 a	0.18 ± 0.00 a	0.53 ± 0.00 b	0.28 ± 0.00 b
	100	1.25 ± 0.01 c	0.82 ± 0.02 c	0.15 ± 0.01 c	0.34 ± 0.00 d	0.31 ± 0.00 a
	200	1.15 ± 0.06 d	0.79 ± 0.02 d	0.13 ± 0.01 d	0.37 ± 0.00 c	0.24 ± 0.00 d
20 October	0	0.91 ± 0.01 c	0.66 ± 0.01 c	0.11 ± 0.00 d	0.70 ± 0.01 b	0.25 ± 0.00 d
	25	1.39 ± 0.04 a	0.84 ± 0.01 b	0.14 ± 0.00 b	0.66 ± 0.01 b	0.34 ± 0.01 c
	50	1.32 ± 0.01 b	0.94 ± 0.01 a	0.14 ± 0.00 b	0.87 ± 0.01 a	0.44 ± 0.00 a
	100	1.29 ± 0.01 b	0.82 ± 0.00 b	0.13 ± 0.00 c	0.34 ± 0.01 c	0.17 ± 0.00 e
	200	1.37 ± 0.04 a	0.94 ± 0.02 a	0.17 ± 0.00 a	0.86 ± 0.05 a	0.38 ± 0.02 b

Data are mean ± standard deviation for $n = 16$. Means with different letters denotes significant differences ($p \leq 0.05$) per harvest date.
